# AB_SA: Accessory genes-Based Source Attribution – tracing the source of *Salmonella enterica* Typhimurium environmental strains

**DOI:** 10.1099/mgen.0.000366

**Published:** 2020-04-22

**Authors:** Laurent Guillier, Michèle Gourmelon, Solen Lozach, Sabrina Cadel-Six, Marie-Léone Vignaud, Nanna Munck, Tine Hald, Federica Palma

**Affiliations:** ^1^​ Laboratory for Food Safety, ANSES, University of Paris-EST, Maisons-Alfort, France; ^2^​ Risk Assessment Department, ANSES, University of Paris-EST, Maisons-Alfort, France; ^3^​ RBE–SGMM, Health, Environment and Microbiology Laboratory, IFREMER, Plouzané, France; ^4^​ Research Group for Genomic Epidemiology, National Food Institute, Technical University of Denmark (DTU), Kongens Lyngby, Denmark

**Keywords:** environmental contamination, multinomial logistic regression, pangenome-wide enrichment analysis, source attribution, *Salmonella *Typhimurium

## Abstract

The partitioning of pathogenic strains isolated in environmental or human cases to their sources is challenging. The pathogens usually colonize multiple animal hosts, including livestock, which contaminate the food-production chain and the environment (e.g. soil and water), posing an additional public-health burden and major challenges in the identification of the source. Genomic data opens up new opportunities for the development of statistical models aiming to indicate the likely source of pathogen contamination. Here, we propose a computationally fast and efficient multinomial logistic regression source-attribution classifier to predict the animal source of bacterial isolates based on ‘source-enriched’ loci extracted from the accessory-genome profiles of a pangenomic dataset. Depending on the accuracy of the model’s self-attribution step, the modeller selects the number of candidate accessory genes that best fit the model for calculating the likelihood of (source) category membership. The Accessory genes-Based Source Attribution (AB_SA) method was applied to a dataset of strains of *Salmonella enterica* Typhimurium and its monophasic variant (* S *. *enterica* 1,4,[5],12:i:-). The model was trained on 69 strains with known animal-source categories (i.e. poultry, ruminant and pig). The AB_SA method helped to identify 8 genes as predictors among the 2802 accessory genes. The self-attribution accuracy was 80 %. The AB_SA model was then able to classify 25 of the 29 *S *. *enterica* Typhimurium and *S *. *enterica* 1,4,[5],12:i:- isolates collected from the environment (considered to be of unknown source) into a specific category (i.e. animal source), with more than 85 % of probability. The AB_SA method herein described provides a user-friendly and valuable tool for performing source-attribution studies in only a few steps. AB_SA is written in R and freely available at https://github.com/lguillier/AB_SA.

## Data Summary

1. The AB_SA (Accessory genes-Based Source Attribution) model is written in R, open-source and freely available from GitHub under the GNU GPLv3 licence (https://github.com/lguillier/AB_SA).

2. All sequencing reads used to generate the assemblies analysed in this study have been deposited in the European Nucleotide Archive (ENA) (http://www.ebi.ac.uk/ena) under project number PRJEB16326. Genome metadata and ENA run accession IDs for all the assemblies are reported in the Supplementary Material (available with the online version of this article).

3. The input data used to carry out the source attribution with the AB_SA method are also available on GitHub (https://github.com/lguillier/AB_SA/tree/master/data).

**Impact Statement**

This article describes AB_SA (Accessory genes-Based Source Attribution), a novel approach for source attribution based on ‘source enriched’ accessory genomics data and unsupervised multinomial logistic regression. We demonstrate that the AB_SA method enables the animal-source prediction of large-scale datasets of bacterial populations through rapid and easy identification of source predictors from the non-core genomic regions. Herein, AB_SA correctly self-attributed the animal source of a set of *Salmonella enterica* Typhimurium and *S *. *enterica* 1,4,[5],12:i:- isolates and further classified the 84 % of strains contaminating natural environments in the pig category (with high probability ranging between ~85 and~99 %).

## Introduction

Tracing the origin of pathogenic microbial strains associated with human diseases or contamination of environmental settings is crucial for identifying targets for intervention in the food-production chain from farm to fork. The process of estimating the probability that human cases or environmental contamination cases arise from putative sources of infection (i.e. animal reservoir, food product and the environment) can be referred to as source attribution.

A variety of methodological approaches has been developed for source attribution of foodborne pathogens: epidemiological approaches (e.g. outbreak data analysis, case–control/cohort studies), microbial subtyping methods, comparative exposure assessment, intervention studies and expert elicitation [1, 2]. In particular, the source-attribution methods based on microbial sub-typing specifically consider genotypic data [1–3].

Genetic variations in micro-organisms are the result of different evolutionary forces. These can be prompted by either neutral processes (genetic drift) or adaptive processes, such as the emergence of a competitively advantageous mutation in a given environment. Most bacterial populations are structured, i.e. their entirety does not form a genetically homogeneous unit, but rather consists of several distinct lineages or sub-lineages that are entirely or partially isolated from one another. Factors such as geographical isolation, combined with random phenomena such as genetic drift and sometimes with local adaptation, drive the genetic differentiation.

Based on genetic targets like a certain number and type of alleles, microsatellites, genes or SNPs, microbial analyses often attempt to infer (sub)population structures identifying the number of clusters, the strains composing them and possible recombination events. Historical methods for studying the genetic relatedness of microbial populations are based on the reconstruction of phylogenetic trees from a matrix of genetic proximities for each pair of strains, typically calculated using the methods proposed by Nei *et al*. [4] or Reynolds *et al*. [5]. Once the tree is built, it can be ‘cut’ at a certain point (e.g. after three levels of nodes from the root) to define the different clusters of strains (more or less equivalent to sub-types). Visually exploring the composition of the clusters (i.e. isolates from different backgrounds) provides a general overview for inferring sources and transmission. However, this approach has been applied rarely in source attribution as inference by phylogeny relies upon the robustness of the tree built on the genetic diversity between isolates, and strains requiring attribution and strains from sources are usually phylogenetically intermixed [6]. Indeed, closely related strains can be found in multiple hosts, challenging the association of a specific source by phylogenetic clustering [7]. A particular case showing the utility of phylogenetic methods in the attribution of human salmonellosis to specific sources (e.g. turkey), by using epidemiological and genomic data, has been reported through the investigation of *Salmonella enterica* Derby genetic diversity [8].

A much different approach relies on the assumption that genetic data (e.g. frequency of different allele numbers at a locus) can be explained by a probabilistic model whose parameters are unknown. Comparing genetic data (frequencies) among different populations allows the establishment of links between strain, e.g. from human cases and different sources. Two structured population genetics models that are currently widely used for source attribution of foodborne diseases are the so-called structure approach [9] and the asymmetric island model (AIM) [10]. These two models are based on different principles of genetic structuring of microbial populations, but the overall attribution approach is similar. These approaches have been successfully applied for source attribution of human sporadic strains for *Campylobacter* spp., [11, 12] *Salmonella* spp. and *Listeria monocytogenes* [13].

Supervised machine-learning approaches are gaining interest in the identification of the causal genetic features associated with the phenotypic traits of microbial pathogens [14], and their use has been discussed for tracing the origin of an outbreak [15] as well. Recent studies also considered such approaches for predicting the source of sporadic human cases [16–18]. In particular, Zhang *et al*. [17] applied a random forest classifier for genomic source prediction. The authors revealed that 50 key genetic features were sufficient for robust source prediction of strains from *S. enterica* Typhimurium. Interestingly, most of these genetic features were accessory genes. Complex phenotypes, such as host adaptation in specific niches, have been linked often to the presence of genes and genetic elements in some strains but not in others (referred to as the ‘accessory genome’), mainly driven by horizontal DNA transfer [7, 19, 20]. Association analysis on a pangenome scale has the potential to relate patterns of genotypic variation (e.g. the differential composition of accessory genes of multi-host lineages) to specific zoonotic niches.

Among supervised classification techniques, multinomial logistic regression (MLR) is a multi-class classification model. It is an extension of binary logistic regression allowing for more than two outcome events. Recently, MLR has been proven to provide a pertinent framework to carry out association analyses across multiple phenotypic traits [21, 22] and in foodborne outbreak investigations as a rule-out tool [23]. The objective of this study was, therefore, to study the performance of MLR in source attribution at the pangenomic scale. In particular, the method was used for predicting to which animal reservoir environmental strains of *S *. *enterica* Typhimurium and its monophasic variant (* S *. *enterica* 1,4,[5],12:i:-) would be attributed to, given the variable set of source-enriched genes from the strains’ accessory genome.

## Methods

### Preliminary genomic analysis

High-quality assemblies of 98 bacterial isolates (see the application to *S. enterica* Typhimurium and its monophasic variant genome dataset section below) were generated by the Technical University of Denmark (DTU) FoodQCPipeline (https://bitbucket.org/genomicepidemiology/foodqcpipeline/src/master/). In short, the FoodQCPipeline trimmed the raw reads using BBDuk2 (part of BBMap v36.49; https://jgi.doe.gov/data-and-tools/bbtools/). Reads were then *de novo* assembled using SPAdes v3.11.05 [24] in the last step of the pipeline. FastQC v0.11.5 (https://www.bioinformatics.babraham.ac.uk/projects/fastqc/) was applied in multiple steps of reads processing (e.g. before and after trimming), generating a quality-control report for each sample. The quality of the *de novo* assemblies was finally assessed using Quast v4.5 [25]. The maximum-likelihood phylogenetic reconstruction of the 98 genome dataset was built on SNPs identified in the core-genome alignments to assess the applicability of the dataset in the study of Munck *et al*. [26]. The annotated tree shows that environmental isolates (i.e. isolates of unknown source) were intermixed with potential sources and, therefore, the dataset is eligible for source attribution (see Data Bibliography; https://itol.embl.de/tree/3758186129366341568123563#) .

### Accessory genes-Based Source Attribution (AB_SA) method

The AB_SA method is based on genomics data. The method is a two-step process. First, the accessory genes enriched in the different sources are calculated (see the preparation of input data for multinomial logistic model pangenome analysis section below). Then, an MLR is developed to predict the probability of animal-source membership for environmental isolates based on ‘source-enriched’ accessory genes.

Thus, multinomial regression is used to explain the relationship between one nominal dependent variable (with more than two levels), that is the source, and one or more independent variables, i.e. the enriched genes. For a source-attribution situation with K sources, the multinomial regression model estimates *k* (k=K−1) from the following equations:

$$Prob(Source=1)=\frac{exp(\beta_{10}+\beta_{11}Gene1+\beta_{12}Gene2+\cdot \cdot \cdot \beta_{1N}GeneN)}{1+exp(\beta_{10}+\beta_{11}Gene1+\beta_{12}Gene2+\cdot \cdot \cdot \beta_{1N}GeneN)}$$

$$Prob(Source=2)=\frac{exp(\beta_{20}+\beta_{21}Gene1+\beta_{22}Gene2+\cdot \cdot \cdot \beta_{2N}GeneN)}{1+exp(\beta_{20}+\beta_{21}Gene1+\beta_{22}Gene2+\cdot \cdot \cdot \beta_{2N}GeneN)}$$

$$Prob(Source=k)=\frac{exp(\beta_{\kappa 0}+\beta_{\kappa 1}Gene1+\beta_{\kappa 2}Gene2+\cdot \cdot \cdot \beta_{\kappa N}GeneN)}{1+exp(\beta_{\kappa 0}+\beta_{\kappa 1}Gene1+\beta_{\kappa 2}Gene2+\cdot \cdot \cdot \beta_{\kappa N}GeneN)}$$

For the final source, the probability of association is derived from the K-1 equations:

Prob(Source=K)=1−(Prob(Source=1)+Prob(Source=2)+⋅⋅⋅+Prob(Source=k))

### Preparation of input data for multinomial logistic model pangenome analysis

The genome assemblies were annotated in (general feature format) GFF3 by using Prokka v1.13.3 [27]. Roary v3.12.0 [28] was used to determine the pangenome of the whole dataset of strains from the annotated assemblies. The dataset included both strains with known (i.e. animal reservoir) and unknown sources (i.e. environmental strains requiring source attribution). To know which genes were enriched in each of the host groups, Scoary v1.6.16 was used [29]. Scoary takes as input the gene_presence_absence.csv file from Roary and a traits file reporting the source associated with each strain. Notice that the traits file is restricted to strains with known sources. The --no_pairwise and --collapse options from Scoary were applied to determine the genes that are enriched in each source. The --no_pairwise flag was used for enrichment analysis to avoid pairwise comparisons and perform a population structure-naive calculation. The --collapse flag was used to merge genes that presented the same pattern of distribution in the sources (a single gene, the first of each merged group of genes was then taken into account by the AB_SA method). The naïve *P* value was used to show the genes most overrepresented in a specific source. A naive threshold *P* value of 0.01 was used to establish the list of potential genes of interest for the attribution of the source, sorted by strength of association per trait. To reduce or increase the number of genes to be considered by the AB_SA method, the user can modify the threshold *P* value.

The CreateInputMNL function from the AB_SA method creates the input files for multinomial logistic model. It takes as arguments: the trait file used as the input of Scoary and the gene presence/absence .Rtab from Roary, and the number of enriched genes to be taken in each source. The function returns two files: a file for fitting the multinomial logistic model on data originating from sources, and a file used for determining the probability of association of a source for unknown strains (i.e. sporadic human strains or environmental strains).

### Training and testing of the multinomial logistic model

Association of sources and genes are then carried out by the multinomial model built on a combination of functions from the ‘nnet’, ‘caret’, ‘e1071’ and ‘ROCR’ R packages [30]. A split-sample approach was applied for training and testing the model using the dataset with known sources to select model tuning values and estimate the model performance through resampling. The dataset with known sources was randomly partitioned into complementary subsets in a ratio of 70 : 30, meaning that 70 % of data will be used for model training and the remaining 30 % for model testing (evaluating model performance) by (K-fold and bootstrapping) resampling method [31]. The resampling method creates modified datasets of samples from the training sets and a model is fit to each resampled dataset to predict the corresponding set of hold-out samples. The aggregation of the results of each hold-out sample set is then used to estimate the resampling performance for finally assessing the more appropriate combination of tuning parameters to consider for the final model refit on the entire dataset. The training and testing are carried out by MNLTrainTest function in AB_SA. It takes as inputs the output file from the CreateInputMNL function, the partition percentage (70 : 30 as default value), as well as the number of bootstraps.

### Assessment of the model’s performance

Trained models were assessed through different accuracy metrics to select the optimal model. The global accuracy of the model, as well as balanced accuracies for each of the sources, were explored. Additionally, to avoid overfitting, the assessment of fitted logistic multinomial models was carried out with regularization. The Akaike information criterion (AIC) was used as a statistical measure of fit to penalize the number of parameters (predictor variables) included in the multinomial logistic model, helping to identify overly complex models that tend to memorize training data. The AB_SA MNLTrainTest function returns both the density plot showing the performance estimates of accuracies and the AIC value.

### Prediction of strains with unknown source

Based on the accuracy values of the trained models and the AIC values, the appropriate number of genes to include in the multinomial logistic model is selected by the modeller. The full set of strains with known sources is fitted with MNLfit function for these genes and samples with unknown source are then predicted using the AB_SA MNLpredict function, which returns a data frame with probability values for each animal source.

### Application to *S *. *enterica* Typhimurium and its monophasic variant genome dataset

The dataset used to explore the relationship between genes of the accessory genome and the animal host was composed of 98 strains belonging to *S *. *enterica* Typhimurium and its monophasic variant collected in 2010–2015. The dataset has been fully described elsewhere [26] and relative epidemiological data is reported in the Supplementary Material. The dataset is composed of strains from known sources and strains with an unknown animal source (i.e. strains requiring attribution). The set of isolates with a known source comprises strains isolated from pigs (*n*=49), poultry (layer chickens, broiler chickens, turkeys and ducks) (*n*=14) and ruminants (cattle, sheep and goats) (*n*=6). For the strains requiring attribution, 29 strains were isolated from the environment (e.g. fresh or brackish water and soil) by ANSES (French Agency for Food, Environmental and Occupational Health and Safety) and IFREMER (French Research Institute for the Exploitation of the Sea), with the collaboration of the University of Caen (France). The IFREMER environmental isolates originated from a research project [32]. They were isolated from freshwater (*n*=12) in Brittany (France) and brackish water in Normandy (France) (*n*=3). The ANSES environmental isolates were isolated from soils (*n*=3) and freshwater (*n*=10). One strain isolated in a crustacean was also associated with this environmental dataset [26].

## Results

### Pangenome-wide enrichment analysis of the *Salmonella* dataset

**Fig. 1.**
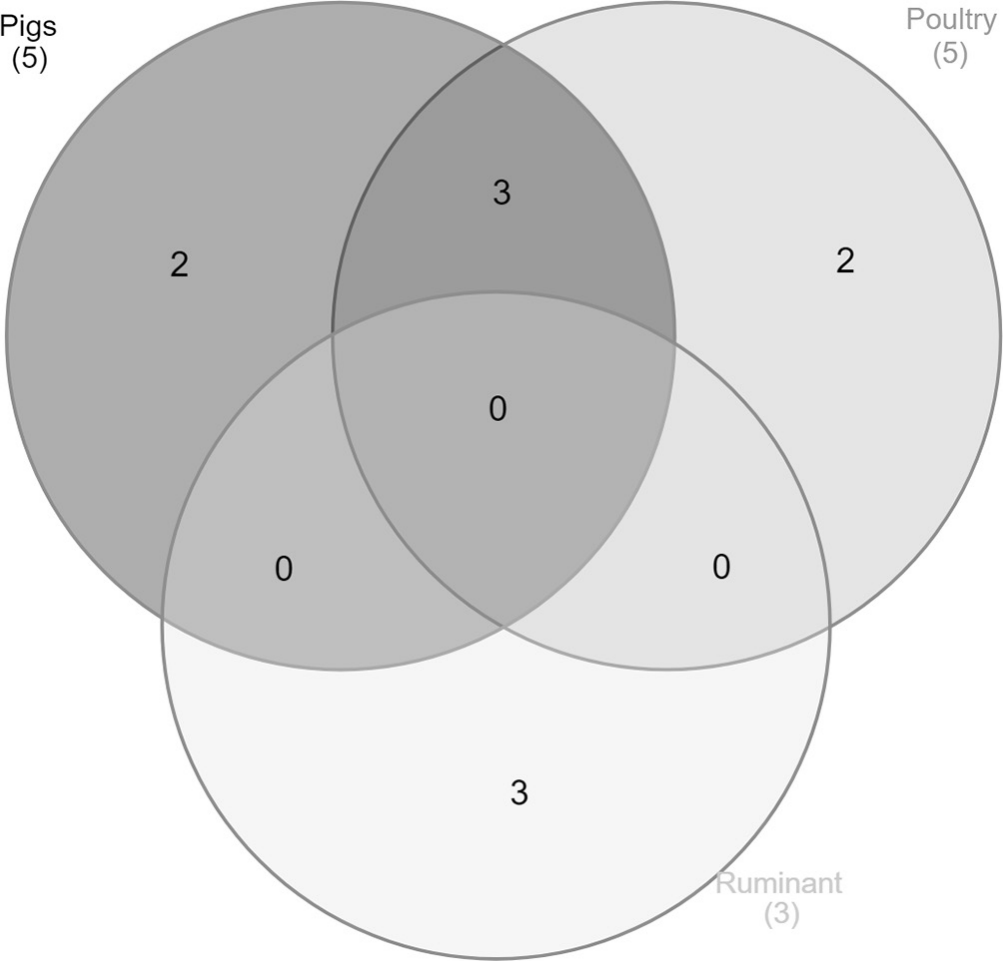
Venn diagram showing the number of source-enriched genes for each animal category. Dark grey, pigs; grey, poultry; light grey, ruminants.

In total, 98 *S *. *enterica* Typhimurium and monophasic variant strains were used in this study as input for implementing an MLR model of source attribution. Of the 98 strains, 29 were isolated from the environment (i.e. water, soil samples and a crab isolate), while the remaining 69 were from animal sources (i.e. pigs, poultry and ruminants). The whole dataset of genomes was used to extract the pangenome, while only the genomes from animal sources were used to score accessory genes as enriched in each animal source. Of the 6988 genes composing the pangenome, 40 % (*n*=2802) represented the accessory genome (present in <99 % of strains). As also reported by Lupolova *et al*. [33], only the differential proportion of genomic variants between their isolation hosts should have predictive value in terms of host restriction and, therefore, source attribution. Here, the whole accessory gene content was considered for the enrichment of genes in the animal sources. With a naive *P* value <0.006, 10 genes were retained as enriched in the selected animal sources (Fig. 1). However, a cluster of four correlated genes was collapsed into a merged unit. Only the first gene of the merged unit together with nine additional ‘source-enriched’ genes will be considered as predictors by the AB_SA model. Most of the candidate genes are specifically enriched in a specific animal source [i.e. ruminants (*n*=3), pigs (*n*=2) and poultry (*n*=2)], while the remaining (*n*=3) are enriched in multiple sources (e.g. pigs and poultry) (Fig. 1).

Feeding the MLR model with the source-enriched genes, a maximum number of genes to consider for predicting the source is arbitrarily selected. In order to select the optimal set of predictors, different numbers of genes (from one to five) were tested, and for each case, accuracy and AIC were assessed (Table 1).

**Table 1.** Tested multinomial logistic models with the accuracy obtained with the training and the AIC values for each selected number of genes

No. of genes/sources	Group_176	Group_763	Group_1926	ymdB_2	ylcG_1	Group_852	Group_6195	Group_160	Group_158	Accuracy [95% CI]	AIC
1	×	–	×	–	–	–	×	–	–	0.82 [0.67,0.91]	92.7
2	×	×	×	–	–	–	×	×	–	0.82 [0.58,0.92]	89.5
3	×	×	×	–	–	–	×	×	×	0.75 [0.64,0.91]	91.2
4	×	×	×	×	–	×	×	×	×	0.74 [0.55,1]	81.2
5	×	×	×	×	×	×	×	×	×	0.71 [0.36,1]	83.6

### Assessment of the multinomial logistic model to predict the strains with known origins

For further performing accurate animal-source prediction, it is necessary to select the gene set that better discriminates pig-, poultry- and ruminant-related genomes. When testing the ability to classify strains with known sources by randomly selecting 70 and 30 % of genomes for training and testing, respectively, global accuracy ranged from 0.71 to 0.82 according to the different genes included in the model (Table 1). Yet the confidence intervals of the accuracies are large, and they could be considered as equivalent. AIC values help to distinguish the best model among those tested. In this study, the model including a total of eight genes as predictors (Table 1) was found to be the best model (with the lowest AIC value). The balanced accuracies obtained with eight predictors are 0.67, 0.70 and 0.9 for pig, poultry and ruminant sources, respectively.

The total number of isolates harbouring these genes and the relative percentage of isolates from each source along with gene annotation from Prokka and the Kyoto Encyclopedia of Genes and Genomes (KEGG) database (https://www.genome.jp/kegg/) are reported in Table 2. Although the functions of most of these genes are not well characterized, some of them were located in prophage/plasmid regions such as *Salmonella* Gifsy-1 (e.g. ymdB_2), Gifsy-2 (e.g. group_176 and group_1926) and a homologue of pRM13516 plasmid (e.g. group_852) from a clinical *Escherichia coli* strain (GenBank accession no. CP006264) (Table 2).

**Table 2.** Predictors of animal sources Numbers of isolates harbouring the predictors with the relative percentage of isolates from the different animal sources and the environment, along with gene annotation from nucleotide and amino acid sequences obtained with Prokka and KEGG, are shown.

Predictor	Total no. of isolates	Pig isolates	Poultry isolates	Ruminant isolates	Env. isolates	Prokka annotation	KEGG protein homology
Group_176	57	0.58	0.05	0.04	0.33	Hypothetical protein	Putative Gifsy-2 prophage protein/DNA breaking–rejoining protein
Group_763	12	0.33	0.58	0.08	0	*rrrD* – lysozyme RrrD	Bacteriophage lysozyme
ymdB_2	12	0.17	0.25	0.25	0.33	Hypothetical protein	Gifsy-1 prophage tail assembly-like protein
Group_1926	6	0	0.67	0	0.33	Hypothetical protein	Gifsy-2 prophage protein/DNA breaking–rejoining protein
Group_852	5	0	0.6	0	0.4	Hypothetical protein	Uncharacterized protein from *E.coli* plasmid pRM13516
Group_6195	3	0	0	0.67	0.33	*cfiA* – 2-oxaloacetate carboxylase large subunit	Oxaloacetate decarboxylase (Na^+^ extruding) subunit alpha
Group_160	8	0.38	0	0.38	0.25	Hypothetical protein	Uncharacterized protein
Group_158	15	0.33	0.2	0.27	0.2	Hypothetical protein	Uncharacterized protein

**Fig. 2.**
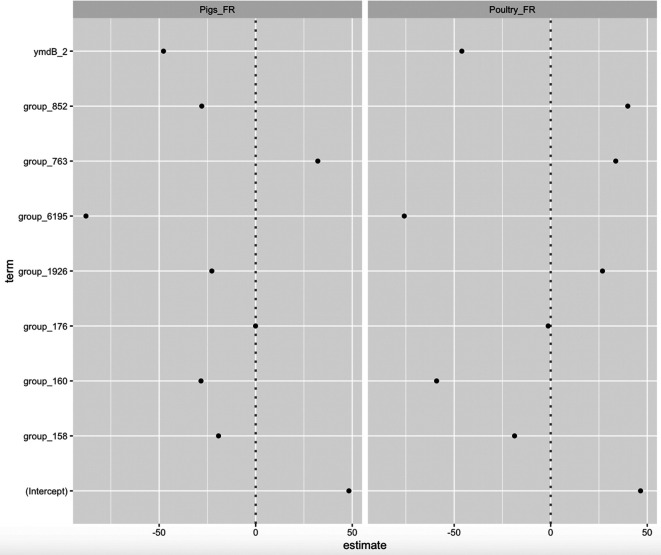
Performance estimates of the parameters of the multinomial logistic model plotting the effect of each predictor. The parameter estimates are relative to the reference category Ruminants_FR. Parameters with significant negative coefficients decrease the likelihood of that response category with respect to the reference category. Parameters with positive coefficients increase the likelihood of that response category.

Therefore, the AB_SA model used this set of ‘best’ predictive genes to classify genomes with unknown sources. The relative importance of each predictor in estimates of the response category (*K-1*) is calculated by the model with respect to the reference category (e.g. Ruminants_FR) (Fig. 2). This statistical measure relates to the weight of each predictor in making a prediction, not whether or not the prediction is accurate. Fig. 2 presents the values of fitted β_κ_ parameters. It shows that some genes have a higher weight than others do. For example, group_6195 presence is strongly associated with ruminants. In the same way, group_852 represents the highest coefficients for poultry.

### Prediction of the origin of environmental strains

**Fig. 3.**
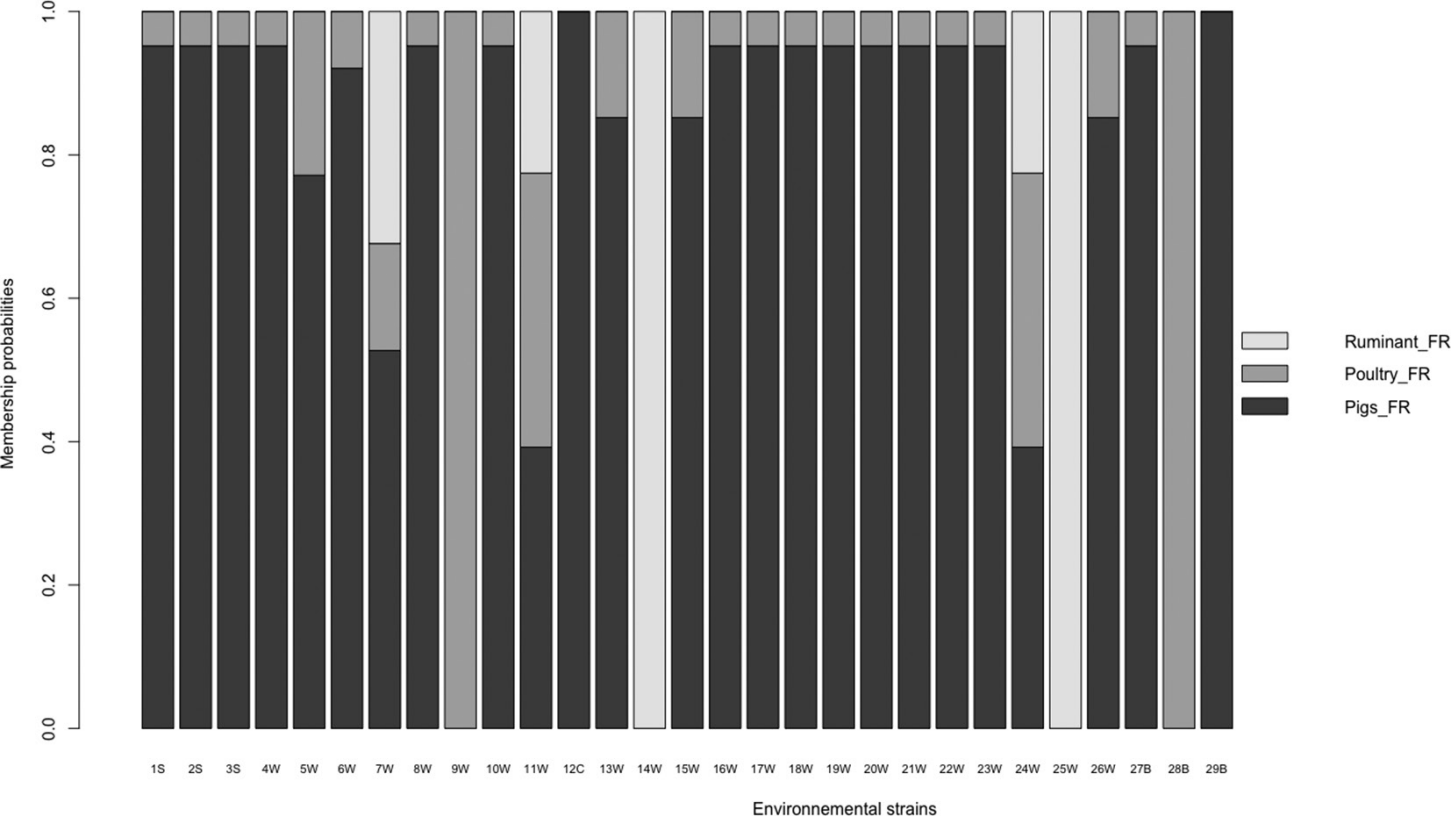
Histogram plot of the individual source-attribution probabilities of the 29 environmental strains of *S *. *enterica* Typhimurium to the three animal sources. The membership probabilities were estimated according to the AB_SA method carried out on the eight source-enriched genes from the accessory genomes. Letters associated with strain numbers refer to the different types of environmental samples: S, Soil; W, fresh water; C, crustacean; B, brackish water.

All the 29 environmental strains were predicted as possible members of the defined animal-source categories with different probabilities. Fig. 3 shows the probabilities of the different environmental strains being associated with one of the three sources. Six strains (i.e. 9, 12, 14, 25, 28 and 29) have a very high membership probability, that is, superior to 99 %, to one of the three sources. The majority of the strains (*n*=19) have a high probability, ranging between 85 and 95 %, of being associated with pig sources. For the four remaining strains (i.e., 5, 7, 11 and 24), the probability of being associated with a specific source is lower than 80 % (e.g. ranging from ~39  to ~77 %). The context of isolation of these strains didn’t provide information that could explain the absence of a strong association for the three sources.

Considering the whole dataset, pig was found to be the most likely source of the strains isolated in the environment.

## Discussion

### Multinomial model for whole-genome-sequencing based source attribution

The environment is not a natural reservoir of *S *. *enterica* Typhimurium. Genomics data are gaining interest in the prediction of the likely source of origin of an isolate [33]. Thus, this study focused on the attribution of 29 *Salmonella* strains isolated from the environment (i.e. river and brackish water, soil and a crab) to potential animal sources based on accessory genes. Recent studies have shown that genetic features from the accessory genomes of *S *. *enterica* Typhimurium isolates constitute a significant signal of host adaptation useful for tracking the source of human strains [7, 17]. Here, the animal sources were grouped into three major categories (i.e. ruminants, poultry and pigs) composed of a dataset of 69 isolates of *S *. *enterica* Typhimurium and its monophasic variant. As in Lupolova *et al*. [18], the SNP-based phylogenomic reconstruction of the 98-genome dataset showed environmental isolates clustered to mixed animal sources (see Data Bibliography), not providing the resolution needed for predicting their possible reservoir. However, genetic factors predictive of animal sources were identified in the accessory genomes of isolates from this dataset through an innovative workflow based on pangenome-wide enrichment and multinomial logistic analysis, the AB_SA model. After the selection of source-enriched genes as predictors, the AB_SA model assesses the probability of a given environmental isolate belonging to each animal source (categorical membership).

MLR-based models are less sensitive to data assumptions (e.g. normality, linearity and homogeneity of data) and have the advantage of limitation of overfitting, which is a common pitfall in machine-learning approaches [34]. Data overfitting occurs when a complex model is trained on too few data points and becomes specific to the training data. As AB_SA returns both accuracy and AIC value (a penalty for model complexity), overfitting is prevented. Moreover, the AB_SA method also returns balanced accuracies for each of the sources. The performance of the trained model with eight predictors was rather similar for the three sources. Balanced accuracy values provide more detailed and appropriate information than global accuracy, especially for unbalanced datasets [35]. As source-attribution studies have to be conducted within a defined period and geographical area, the compilation of a large dataset is a challenge [2]. Logistic regression methods require less data than other classification methods like random forest to achieve stability [36]; the AB_SA method is thought to be appropriate for many source-attribution studies.

AB_SA is flexible in the choice of the level of significance (through a threshold value for the *P* value) of enriched genes and in the number of candidate predictors per source to feed the multinomial logistic model. This MLR model also provides a measure of the weight of each predictor (i.e. gene) for each source category, although the interpretation of the coefficients is not immediate. The interpretation might be further complicated by not having a single set of coefficients, but as many sets as the number of sources minus one (*K-1*).

A common parameter to assess the success of source-tracing models is the self-attribution accuracy, usually calculated by training the model on split known datasets (e.g. 30 % testing and 70 % training). Consistent with published source-attribution studies [12, 17, 18], the overall self-attribution accuracy, as well as the source balanced accuracies of this study, were 67–90 % . Similar results were also observed in a recent review [33], where different supervised ML methods (e.g. single vector machine, random forest and neural network) achieved ~80 % accuracy in the prediction of the bacterial source of isolation.

### Number of genes predictive of animal source

The enrichment step of the AB_SA method allowed us to select the most relevant genes for modelling their effects with the multinomial logistic model. Within the 2802 accessory genes, 8 genes were finally selected in the best model for the prediction of the origin of environmental strains. The number of genes identified for source attribution of *S *. *enterica* Typhimurium in the USA was larger [15]. Yet the number of sources considered were higher and the larger dataset might improve statistical power and the ability to identify additional pertinent predictors.

The greater number of predictors used in the training phase improved the model's performance. However, using more than eight genes as predictors returned a worse AIC value in this dataset, as the use of more predictors led to overfitting in the regression. This observation shows that high dimensional input data for source-attribution models do not guarantee high performance. The same finding has been observed with source attribution based on the core genome. In a source-attribution study for *L. monocytogenes *, Nielsen *et al*. [13] observed that similar accuracy values can be achieved feeding multilocus sequence type (MLST)-based source-attribution models with seven loci or thousands of loci (core-genome MLST). Population models like the structure model or the asymmetric island model (AIM) are still pertinent approaches for source attribution with genomics data. Yet, rather than increasing the number of loci, the selection of pertinent genes to be used as input can be done. Recently, this approach has been applied for *Campylobacter *: high accuracy was obtained with 15 host-segregating genetic markers used as inputs of the structure model [37].

### Source prediction of strains using accessory genes as predictors

In this study, eight genomic factors making up the ancillary genomes of the observed dataset were selected as predictive of animal sources (Table 2; sequences are available in the Supplementary Material) by the AB_SA method. Although several hypothetical proteins were present, the selected genes had different putative functions, including structural function (e.g. the *cfiA* gene involved in membrane protein catalysis for ATP synthesis, transport and motility) [38], as well as DNA packaging and lysis (e.g. DNA breaking-rejoining protein, lysozyme, prophage tail assembly protein) (Table 2).

Interestingly, the majority (*n*=7/8) of these predictors were located in mobile genetic element regions, such as putative prophages and plasmid elements. The plasmid element harbouring the predictor does not seem to be related to any publicly available *S *. *enterica* Typhimurium sequence; rather, it seems a remnant plasmid homologous to *E. coli* plasmids including genes encoding DNA transfer functions (e.g. type IV conjugative transfer protein TraL). The presence of unique accessory genomic biomarkers within prophage regions such as Gifsy-1 has been shown recently to contribute to enhancement of the ability to trace back the origin of epidemic clones of *S *. *enterica* Typhimurium and its monophasic variant on a geographical scale [39]. Here, half of the animal-source genomic markers identified in the assessment of the probability of correct self-attribution were prophage-related genes, suggesting that prophage elements might play a crucial role in source-tracking studies also. As observed by Zhang *et al*. [17], plasmid- and prophage-related loci may constitute highly informative predictors of livestock sources of *S *. *enterica* Typhimurium and, therefore, contribute to the optimization of source-attribution models for surveillance or outbreak investigations.

The importance of accessory genes in host adaptation is not limited to *S *. *enterica* Typhimurium. Recent studies focused on the pangenome of *Campylobacter jejuni* showed that host-segregating genomic factors located in the accessory genomes constitute epidemiological markers for source attribution [12, 40].

### Tracing the source of the *Salmonella* spp. environmental strains

*Salmonella* spp. strains are frequently detected in surface-water samples, e.g. 30.1 % of samples from three French coastal catchments [32], 43 % of samples from Georgia (USA) [41] and 23 % of samples from Canada [42]. In particular, *S *. *enterica* Typhimurium and its monophasic variants were isolated more frequently in some sites than others [32]. Pigs, poultry and ruminants (e.g. cattle) constitute a relevant source of these pathogens acting as natural salmonella reservoirs, without showing any symptoms while shedding into the environment [43]. In this study, pigs were found to be the most likely source of contamination for most of the *S *. *enterica* Typhimurium and *S *. *enterica* 1,4,[5],12:i:- strains isolated in the environment. This result confirms observations from several source-tracking studies that pointed out some associations between environmental *Salmonella* spp. strains and pig production [42–44]. This is also consistent with the fact that pigs are asymptomatic carriers of *S *. *enterica* Typhimurium, generating a major reservoir for salmonella strains, which contaminate the environment around the primary production sites [45, 46].

Although waterborne outbreaks of *S *. *enterica* Typhimurium have been reported rarely, a secondary waterborne outbreak in a rural community has been linked to *S *. *enterica* 1,4,[5],12:i:- and the leaching of animal faecal matter into groundwater destined for human consumption [47]. Also, anthropological activities such as fertilization with animal manure and irrigation with water contaminated by livestock and/or wildlife faecal matter seem likely to be involved in salmonella outbreaks linked with the consumption of fresh produce [48, 49]. Gaining insights into the animal sources of strains contaminating natural environments is, therefore, of great importance for providing evidence to support targeted interventions and policy development to reduce the public-health risk. Critical factors such as the limited number of available genomes (unrepresentative of the whole population structure) and the absence of evidence on possible transmission/contamination points are a challenge to source attribution. However, the combination of pangenomic analysis and the supervised MLR model applied to our dataset succeeded in identifying genetic signals associated with different animal sources, helping to predict the likely reservoir of isolates from the natural environment. The capacity to predict the likely reservoir of an isolate using source-attribution models such as AB_SA can be relevant for understanding not only the contamination of a water source but also the origins of an outbreak, humans or food-product isolates.

## Data Bibliography

1. Munck N *et al*. Assemblies used in this study have been deposited in the ENA (http://www.ebi.ac.uk/ena) under project number PRJEB16326 (2019).

2. Palma F. Phylogenetic reconstruction of the studied dataset with isolate metadata for the 98 strains can be viewed on iTOL: https://itol.embl.de/tree/3758186129366341568123563# (2019).

3. Guillier L. Input data. (enriched genes in sources) used in the paper of well as AB_SA scripts are available on GitHub and the associated Zenodo repository, DOI:10.5281/zenodo.3507204 (https://zenodo.org/record/3507204#.XpB7SHJKiM8) (2019).

4. Munck N *et al*. The machine-accessible metadata file describing the four European *Salmonella enterica* Typhimurium datasets collected to develop the whole-genome-sequencing based source-attribution methods is available on Figshare, https://doi.org/10.6084/m9.figshare.c.4748825 (2020).

## Supplementary Data

Supplementary material 1
